# Stability-Guided Formulation of a Light-Sensitive D-LSD Capsule for Clinical Investigation

**DOI:** 10.3390/pharmaceutics17060767

**Published:** 2025-06-11

**Authors:** Bernard Do, Luc Mallet, Maxime Annereau, Danielle Libong, Audrey Solgadi, Florence Vorspan, Muriel Paul, Philippe-Henri Secretan

**Affiliations:** 1 Clinical Pharmacy Department, Gustave Roussy Cancer Campus, 94805 Villejuif, France; 2 Institut des Sciences Moléculaires d’Orsay, CNRS, Université Paris Saclay, 91400 Orsay, France; 3 Université Paris-Est Créteil, DMU IMPACT, Département Médical-Universitaire de Psychiatrie et d’Addictologie, Hôpitaux Universitaires Henri Mondor-Albert Chenevier, Assistance Publique-Hôpitaux de Paris, 94000 Créteil, France; 4 Sorbonne Université, Institut du Cerveau-Paris Brain Institute–ICM, Inserm, CNRS, 75013 Paris, France; 5 Department of Mental Health and Psychiatry, Global Health Institute, University of Geneva, 12011 Geneva, Switzerland; 6 Lip(Sys)2—Chimie Analytique Pharmaceutique, Université Paris-Saclay, 91400 Orsay, France; 7 UMS-IPSIT SAMM Facility, Université Paris-Saclay, Inserm, CNRS, Ingénierie et Plateformes au Service de l’Innovation Thérapeutique, 91400 Orsay, France; 8 Université Paris Cité, INSERM UMRS 1144 Optimisation Thérapeutique en Neuropharmacologie, 75006 Paris, France; 9 APHP, GHU NORD, Hôpital Fernand Widal, Département de Psychiatrie et de Médecine Addictologique, 75010 Paris, France; 10 Department of Pharmacy, Henri Mondor Hospital, AP-HP, 94000 Créteil, France; 11 Matériaux et Santé, Université Paris-Saclay, 91400 Orsay, France

**Keywords:** D-LSD, photostability, formulation development, orthogonal analysis, LC-IM-MS, DFT, degradation products

## Abstract

**Background/Objectives:** D-lysergic acid diethylamide (D-LSD) is under investigation as a potential therapeutic strategy for alcohol use disorder (AUD). However, the extreme light sensitivity of D-LSD presents a significant challenge in developing suitable pharmaceutical forms, particularly for clinical trial settings. This study proposes a liquid-filled capsule formulation designed to provide accurate dosing while protecting D-LSD from photodegradation. **Methods:** To support formulation development and ensure its suitability as an investigational medicinal product, a multi-tiered analytical strategy was employed. This included liquid chromatography coupled with ion mobility spectrometry and mass spectrometry (LC-IM-MS), along with quantum chemical calculations (density functional theory (DFT) and time dependent-DFT (TD-DFT)), to ensure robust and orthogonal structural characterization of degradation products. **Results:** Photostress studies demonstrated that while D-LSD in solution rapidly degrades into photoisomers and photooxidative byproducts, the capsule formulation markedly mitigates these transformations under ICH-compliant conditions. **Conclusions:** These findings highlight the essential role of orthogonal stability profiling in guiding formulation development and demonstrate that this approach may offer a viable, photostable platform for future clinical investigation of D-LSD in the treatment of AUD.

## 1. Introduction

Alcohol use disorder (AUD) is a major public health concern, with limited therapeutic success in patients suffering from severe dependence. Despite the availability of pharmacological and psychotherapeutic treatments [1], relapse rates remain high [2]. Recent preclinical and historical clinical studies suggest that lysergic acid diethylamide (LSD), a classic serotonergic psychedelic, may offer a novel therapeutic approach for AUD. LSD exerts its effects primarily through agonism at the serotonin 5-HT2A receptor, a target implicated in both affective regulation and substance use disorders, and has been shown to reduce ethanol intake in animal models [3,4]. Furthermore, clinical investigations conducted in the mid-20th century reported improved abstinence rates following high-dose LSD administration in individuals with alcohol dependence [5,6]. To further evaluate these findings, a multicenter clinical trial is currently being initiated in France to assess the efficacy of single oral doses of D-LSD in patients with AUD.

However, before such clinical investigations can proceed, a key pharmaceutical challenge must be addressed: D-LSD is highly sensitive to photodegradation, oxidation, and other environmental stressors, leading to the formation of potentially active or undesirable degradation products such as iso-LSD, lumi-LSD, and various oxidative derivatives [7,8,9]. To enable clinical evaluation in protocols, a formulation must therefore ensure (i) protection from environmental triggers, (ii) dose accuracy and reproducibility, and (iii) chemical stability over the intended shelf-life and administration window. To address these requirements, we developed a liquid-filled capsule formulation designed to minimize degradation risks through a combination of non-aqueous solvation, viscous matrix stabilization, and physical light shielding.

While conventional LC-UV-MS approaches are widely used for pharmaceutical stability testing and have been broadly applied to quantify D-LSD in seized drugs [10,11] and biological matrices [12,13], they often fail to resolve structurally similar degradation products, particularly photoisomers [14], which are common in D-LSD photochemistry. In this context, we implemented a photostability and degradation profiling study using a pharmaceutical relevant light source (complying with ICH Q1B recommendations [15]), orthogonal analytical techniques (liquid chromatography coupled to ion mobility–mass spectrometry [16]), and quantum chemical simulations to evaluate the behavior of D-LSD in solution and within the formulation.

Beyond characterizing D-LSD photodegradation products, a key objective of this study was to ensure that the novel non-aqueous formulation does not introduce new degradation pathways. This approach is critical from a safety and regulatory standpoint, particularly considering existing LSD-based formulations that rely on hydroalcoholic matrices. Our findings provide a robust analytical foundation for the stability and integrity of the liquid-filled capsule formulation under photostress and support its advancement as a promising platform for the clinical evaluation of D-LSD in AUD.

## 2. Materials and Methods

### 2.1. Materials

D-LSD tartaric salt (D-Lysergic acid diethylamide, D(-)-Tartaric acid, purity > 98.5%) was purchased from Lipomed (Arlesheim, Switzerland). Pharmaceutical grade excipients, complying with Ph. Eur. Monograph requirements, polysorbate 80 and sodium acetate trihydrate, and meeting USP specification, propylene glycol, as well as well as analytical grade solvents, acetonitrile and methanol, were obtained from Merck (Fontenay-sous-Bois, France).

### 2.2. Capsules Preparation and Sample Preparation

Relative composition of the liquid fill and quantities for a 100 µg capsule are reported in Table 1.

To prepare the liquid fill, the required amount of D-LSD and excipients are accurately weighed using a precision balance. The solid ingredients and the liquid excipients are then successively placed in a 50 mL clean Pyrex^®^. The mixture is then mixed using a stirring bar until complete dissolution is achieved.

For photostability studies in solution, a methanolic stock solution (100 µg.mL^−1^) was diluted in ultra pure water to reach a final concentration of 25 µg.mL^−1^ prior to exposure to ICH Q1B conditions.

To study the photostability of D-LSD in the liquid fill (1280 µg.mL^−1^), the fill was exposed to ICH Q1B and then diluted in ultra pure water and methanol (50/50) to reach a final concentration of 25 µg.mL^−1^.

### 2.3. Instrumental

The analytical conditions described in this study were developed to characterize degradation products of D-LSD under photostress. They were optimized for selectivity, peak resolution, and spectral identification, rather than for quantitative assay. A separate, fully validated assay method for D-LSD content determination has been developed and is included in the IMPD but is not within the scope of this manuscript

#### 2.3.1. Liquid Chromatography

Chromatographic separations were achieved using a Nexera-UC (Shimadzu, Kyoto, Japan) chromatographic system comprising 2 LC-40DXR UHPLC pumps, a SIL-40CXR autosampler, and a SPD-M40 photodiode array detector controlled via LabSolution^®^ software version 5.110 (Shimadzu). Separations were carried using a Kinetex (Phenomenex, Torrance, CA, USA) column (150 nm × 4.6 nm; 5 µm), maintained at 30 °C. The injection volume and flow rate were set at 1 mL min^−1^ and 50 µL, respectively. The gradient program was set as follows: 0–2 min: 95%A; 2–30 min: 95 → 0% A; 30–35 min: 0, where A = [water (99.9%) + formic acid (0.1%)] and B = [acetonitrile (99.9%) + formic acid (0.1%)]. The flow rate entering the mass spectrometer was decreased down to 0.3 mL min^−1^ using a 1/3 T split. Chromatograms were recorded at 220 nm.

#### 2.3.2. Ion Mobility and Mass Spectrometry Conditions

Mass spectra were acquired using a timsTOF Pro 2 Q-TOF mass spectrometer equipped with trapped ion mobility technology (Bruker, Bremen, Germany). Analysis was carried out in positive ion mode (ESI^+^) as per the following conditions: capillary voltage and end plate offset was set respectively at 4500 and 500 V, nebulizer pressure was set at 2.2 bar, dry gas at 8 L.min^−1^, and dry temperature at 200 °C.

Ion transfer parameters were 60 V for deflection 1 delta, 250 Vpp for funnel 1 RF, 200 Vpp for funnel 2 RF, and 200 Vpp for multipole RF. Trapped ion mobility separation (TIMS) were as follows: 1/k_0_ range between 0.45 and 1.54 V.s.cm^−2^, ramp time of 300 ms, and ICC target 7.5 Mions. Ion fragmentation was performed using PASEF (parallel accumulation serial fragmentation) mode including 2 MS/MS scans with a collision energy of 20 eV, an isolation width of 2–8 *m*/*z* on a mass range of 20–1000 *m*/*z*, a target intensity of 4000, and an intensity threshold of 100. Other detailed parameters are provided in Table S1.

The MS data were processed using Data Analysis^®^ software (version 6.1 build 119.2.0).

#### 2.3.3. Photostability Conditions

The solutions and capsules were exposed to artificial weathering light, using a xenon test chamber Q-SUN Xe-1 operating in window mode. This light source meets the requirement of light sources (option 1) provided in ICH Q1B [11]. Indeed, it is designed to produce an output similar to the D65/ID65 emission standard, the internationally recognized standard for outdoor daylight. 

The light beam, presenting a characteristic spectrum ranging from 300 to 800 nm, was delivered at an intensity of 1.50 W/m^2^ at 420 nm (UV irradiance from 300 to 400 nm: 66.5 W/m^2^; illuminance: 119.6 klx). The temperature was set at 25 °C.

### 2.4. Theoretical Calculations

All structures investigated in the manuscript were subjected to conformational analysis employing the OPLS4 force field implemented in the MacroModel program (MacroModel Schrödinger Release 2022-1: MacroModel; Schrödinger LLC: New York, NY, USA, 2022) followed by geometry optimization on each of the lowest energy conformers. Frequency calculations were performed to ensure that the geometries represented a true minimum (i.e., no imaginary frequencies).

The spin densities of D-LSD and the highest occupied molecular orbitals (HOMO) were visualized by mapping their values to the electron density surface. The UV-Vis absorption spectra were obtained using time-dependent density functional theory (TD-DFT) calculations, performed with the Schrödinger Materials Science Suite. The simulated UV-Vis spectra were generated based on the calculated excitation energies and oscillator strengths of the 20 first excited states of each studied compound. Excited state energy calculations were performed using the excited state analysis module as implemented in Maestro (Schrödinger LLC: New York, NY, USA, 2022).

All calculations were performed using the B3LYP exchange–correlation functional with the D3 a posteriori correction, together with the 6-31G++** basis set.

## 3. Results and Discussion

### 3.1. Formulation Strategy

The design of a pharmaceutical formulation for D-LSD involves several critical challenges, including the compound’s **extreme environmental sensitivity**, the need for **precise microgram-level dosing**, and ensuring **stability over shelf-life and use**. D-LSD is known from the literature to be highly susceptible to **photodegradation**, **oxidation**, and **acid- or base-catalyzed transformations**, particularly when in dilute aqueous solutions or exposed to light and air.

These known vulnerabilities guided the formulation strategy toward minimizing the drug’s interaction with light, moisture, and oxygen. To achieve this, a **non-aqueous liquid system** based on **polysorbate 80 and propylene glycol** was selected. This matrix offers several stabilizing features:

**Low water content**, to reduce hydrolysis and limit oxygen solubility;

**Viscous consistency**, which restricts molecular motion and may suppress degradation kinetics;

**Surfactant and co-solvent properties**, promoting full dissolution of D-LSD and limiting surface adsorption, crucial for dose reproducibility at microdose levels.

This solution-based fill system was favored over a suspension to ensure **homogeneity and content uniformity** and to enable accurate dosing in **unit-filled hard-gelatin capsules**. The low water activity and vehicle compatibility also allow the use of standard capsule shells without compromising their physical integrity.

In addition to these formulation-level considerations, the entire dosage unit was designed with a **multi-tiered protection strategy**, including **opaque capsule shells** and **amber glass vials**, intended to shield the drug from light and oxidative environments during storage and handling. Regarding the presence of polysorbate 80 (56 mg per unit dose) and propylene glycol (8 mg per unit dose), though their amounts do not exceed the thresholds provided in EMA guidance [17,18] (25 and 50 mg/kg body weight/day, respectively), the mandatory information regarding them will be included in the package leaflet.

Subsequent analytical studies (Section 3.2) were conducted to assess the photostability of this formulation and verify whether it successfully mitigates the degradation risks anticipated from D-LSD’s known reactivity.

### 3.2. Characterization of D-LSD Degradation in Solution upon Light Exposure

Given the well-documented light sensitivity and environmental instability of D-LSD, establishing the stability of the active substance within the chosen pharmaceutical form was essential. While conventional stability testing focuses primarily on assay and impurity thresholds, a more detailed investigation into the nature and identity of degradation products (DPs) was deemed necessary to ensure the safety and pharmaceutical quality of the formulation. This is particularly important in the context of investigational use, where the therapeutic index may be narrow and where degradation products—especially photoisomers and oxidized derivatives—may exhibit unexpected pharmacodynamic or toxicological profiles.

To address this, we conducted a rigorous photostability study using both conventional and orthogonal analytical techniques, not only to monitor the degradation kinetics of D-LSD in solution but also to structurally characterize the resulting DPs. This deep characterization enabled the definition of a reference degradation profile in unformulated solution. Importantly, subsequent analysis of the capsule formulation confirmed that no additional or novel degradation products were generated as a result of interactions with excipients. This provides strong evidence that the excipient matrix does not induce alternative degradation pathways beyond those already known for D-LSD, supporting the pharmaceutical robustness and clinical suitability of the final formulation.

To determine the structures of the degradation products formed during simulated light exposure, a stock solution of D-LSD was subjected to photostress in a calibrated light chamber meeting ICH Q1B requirements. The exposed samples were analyzed via liquid chromatography coupled to diode array detection (LC-DAD) and ion mobility-time-of-flight mass spectrometry (LC-IM-TOF), allowing multidimensional detection and structural insight. Exposure to these conditions resulted in the formation of several degradation products, as evidenced by distinct peaks in both chromatographic and ion mobility profiles. These included signals consistent with known photoisomers, such as iso-LSD, and an additional compound tentatively identified as an oxidative derivative, referred to as DP2. The ion mobility separation proved essential for resolving co-eluting isomers, while UV spectra and high-resolution MS data supported the preliminary structural assignments. The resulting profile serves as a benchmark for comparison with the behavior of the encapsulated formulation under equivalent stress conditions.

#### 3.2.1. Degradation Products Detected via LC-UV, LC-MS, and LC-IM-MS

**Figure 1:**
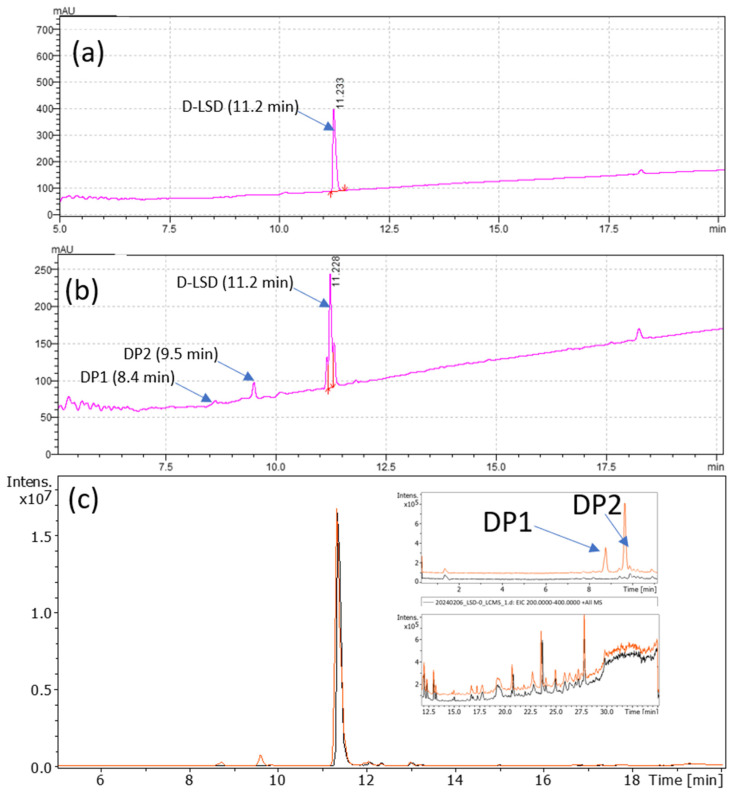
LC-UV and LC-MS analysis of D-LSD solution before and after light exposure; (**a**) LC-UV chromatogram of freshly prepared D-LSD solution, showing the main peak at 11.2 min under baseline conditions; (**b**) LC-UV chromatogram of the same solution after 30 s of ICH Q1B-compliant photostress exposure, revealing the appearance of two new peaks at 8.4 and 9.5 min; (**c**) overlay of total ion chromatograms (TIC) before (black) and after (orange) light exposure, highlighting the formation of degradation products in the exposed sample.

Photostressed D-LSD solutions were analyzed using liquid chromatography coupled with UV detection (LC-UV) and mass spectrometry (LC-MS). As shown in Figure 1, exposure to light under ICH Q1B conditions resulted in the appearance of two additional peaks at 8.4 min (DP1) and 9.5 min (DP2), respectively, in both the UV and total ion chromatograms. These peaks were absent in freshly prepared samples, where LSD elutes at 11.2 min, and are attributed to photoinduced degradation products.

**Figure 2:**
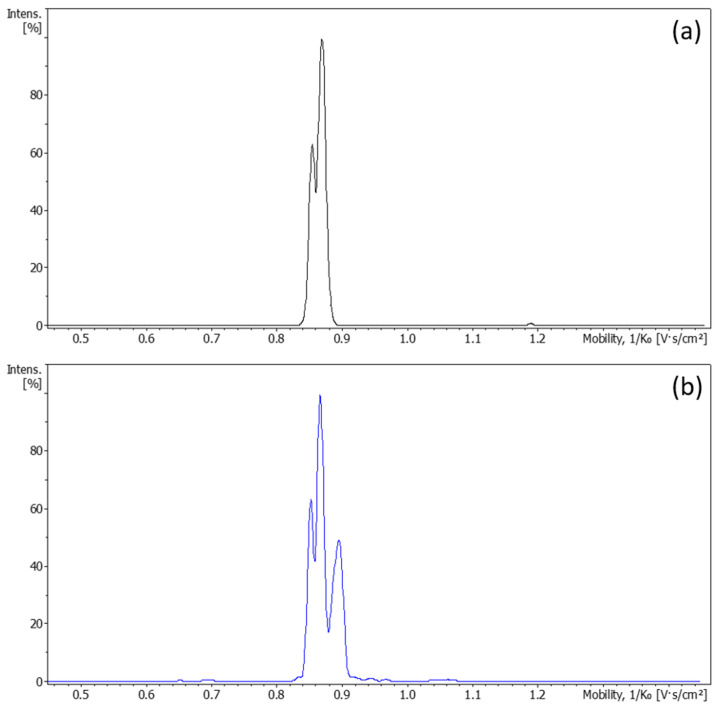
Ion mobility separation of isomeric species in D-LSD standard and photostressed samples; (**a**) extracted mobilogram for *m*/*z* 324.2072 in the standard D-LSD solution, showing two conformers (protomers) corresponding to the native compound; (**b**) extracted mobilogram after light exposure, revealing a new drift time feature (0.88–0.90) consistent with a photoisomer of D-LSD (tentatively assigned as LSD-iso). This figure demonstrates the ability of LC-IM-MS to resolve isomeric degradation products that co-elute in conventional LC.

It is well known that upon light exposure, LSD can form isomeric counterparts, some of which may co-elute under reverse-phase LC conditions. To assess whether such co-elution occurred, extracted mobilograms of the *m*/*z* 324.2072 ion were recorded for each main chromatographic peak using LC-IM-TOF analysis. The mobilogram of the standard solution recorded at the retention time of the D-LSD revealed two distinguishable ion mobility features corresponding to LSD protomers (Figure 2a). Upon light exposure, at the retention time of D-LSD a third isomeric species emerged, characterized by a mobility between 0.88 and 0.90 (Figure 2b). The relative peak height of this feature increased from 15% to 47% when the solution was exposed to respectively 30 s and 120 s of simulated light, confirming the photoinduced formation of an isomer, herein referred to as LSD-iso.

These findings confirm the generation of isomeric degradation products of D-LSD in solution under photostress, with distinct chromatographic and ion mobility characteristics. The combination of LC-UV, LC-MS, and LC-IM-MS techniques provided a multidimensional fingerprint for DP1 and DP2. This analytical profile serves as a benchmark for assessing the protective effect of the capsule formulation, described in subsequent sections.

#### 3.2.2. Tentative Structural Characterization of the Main Photodegradation Product: Interpretation and Converging Evidence

Photostress studies revealed two consistent degradation products of D-LSD in solution: DP1 (RT 8.4 min) and DP2 (RT 9.5 min). These species were clearly resolved from each other and from parent D-LSD (RT 11.2 min), both chromatographically and spectrally. Among them, DP2 emerged as the dominant and most structurally tractable photoproduct, displaying UV absorbance features distinct from D-LSD and reproducible across replicates.

High-resolution MS established that DP2 exhibits a mass of *m*/*z* 342.202 [M+H]^+^, consistent with the elemental formula C_20_H_28_N_3_O_2_^+^. This +18 Da mass shift relative to D-LSD (C_20_H_26_N_3_O^+^, *m*/*z* 324.207) indicates photooxidation, rather than isomerization.

To explore its structural origin, DFT energy calculations were performed to simulate the excited-state behavior and photooxidation pathways of D-LSD. The low-lying excited singlet (S_1_ = 4.14 eV) and triplet (T_1_ = 2.45 eV) states support the feasibility of intersystem crossing and energy transfer to triplet oxygen, yielding singlet oxygen (^1^O_2_). Moreover, the strongly negative oxidation potentials at both excited states (−1.98 eV and −1.39 eV) confirm that photoinduced oxidation is thermodynamically favored.

**Figure 3:**
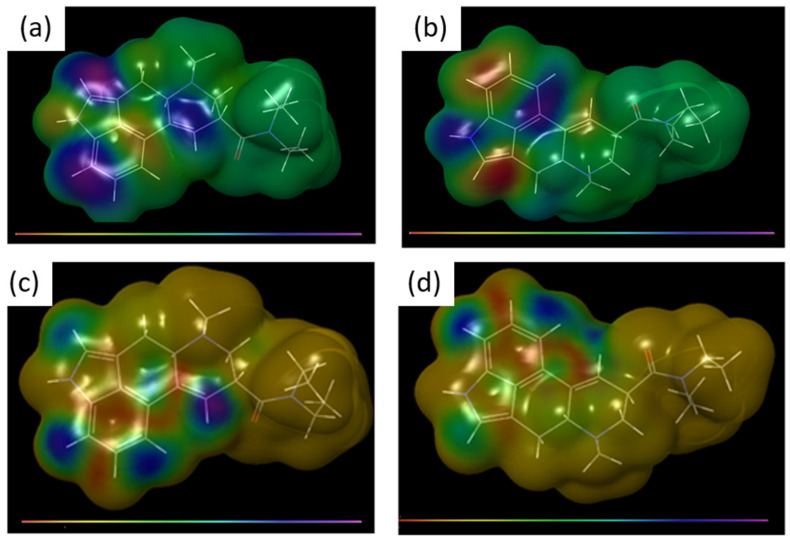
Spin density and HOMO mapping of the D-LSD radical cation (D-LSD^•+^); (**a**,**b**) calculated HOMO electron density map of D-LSD^•+^ showing localization on the indole nitrogen and C8=C9 double bond, suggesting likely sites for oxidation; (**c**,**d**) spin density map of D-LSD^•+^, highlighting reactive regions with potential to initiate photochemical transformation. Regions with the lowest and highest values are mapped in red and purple, respectively (see color scale). These calculations support the mechanistic plausibility of oxidative degradation pathways leading to DP2.

To predict where such oxidation might occur, we analyzed the highest occupied molecular orbital (HOMO) and spin density of the D-LSD radical cation (D-LSD•^+^). Both maps revealed high electron and spin localization around the indole nitrogen and the C8=C9 double bond (Figure 3), pinpointing the most reactive regions likely to initiate chemical transformation.

**Figure 4:**
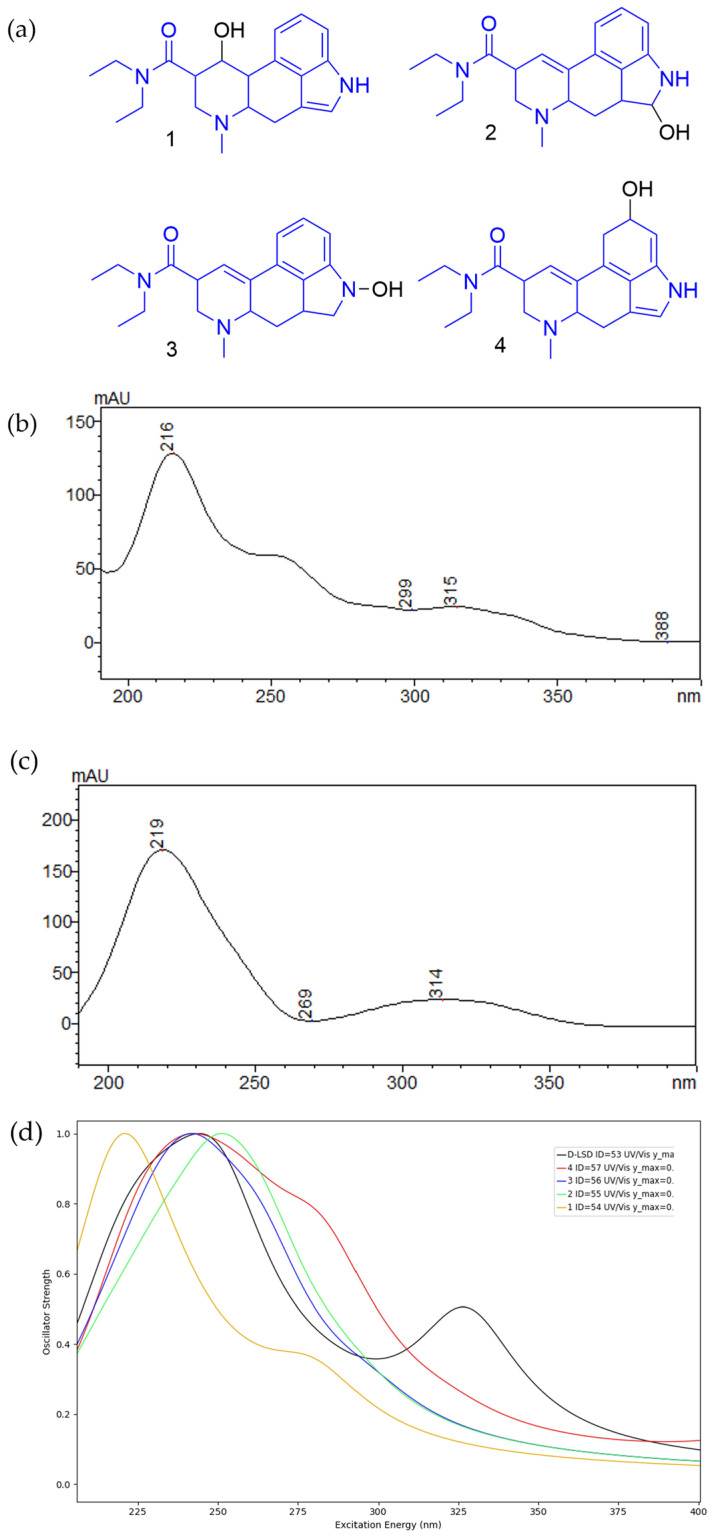
TD-DFT-predicted and experimental UV spectra for candidate DP2 structures; (**a**) structures of four plausible oxidative degradation products derived from D-LSD spin density localization; (**b**) experimental LC-DAD UV spectrum of DP2 eluting at 9.5 min after light exposure; (**c**) UV spectrum of standard D-LSD; (**d**) TD-DFT-simulated spectra for the candidate structures shown in (**a**). Only Compound 1 (brown trace) matches the experimental DP2 spectrum, especially the shoulder at ~275 nm. This supports assignment of DP2 to a specific oxidative rearrangement product.

Based on this, four structurally plausible photooxidation products were proposed (Figure 4, inset (a)). To evaluate which best matched DP2, TD-DFT UV spectra were simulated for each candidate. Among these, Compound 1 reproduced the key spectral features observed experimentally for DP2, including a distinct shoulder at ~275 nm in the LC-DAD trace (Figure 4, inset (b) vs. (d)). This spectral convergence provides strong support for the assignment of DP2 as a photooxidized D-LSD derivative, formed via singlet oxygen addition or radical oxidation at the ergoline system. The proposed structure was further verified via LC-IM-MS^2^-based studies detailed in the Supplementary Information (Figures S1–S5).

In contrast, DP1, though detected as the second major degradation product, remains structurally unresolved. It is isobaric with D-LSD (*m*/*z* 324.207) and elutes earlier (RT 8.4 min). Most notably, DP1 shows a shorter ion mobility drift time (~0.885, Figure S6) than the two protomers of D-LSD (0.85–0.88), indicating a less compact gas-phase conformation. These features suggest an isomeric transformation, such as formation of iso-LSD or a related photorearrangement.

However, none of the TD-DFT simulated candidates tested reproduced the spectral features of DP1. This may indicate an alternative mechanism, such as tautomerization, cis–trans isomerization, or the involvement of a chromophore configuration not captured by the initial modelling approach. Despite this, the consistent presence, mobility separation, and distinct UV features of DP1 confirm that it arises via a mechanistically distinct pathway from DP2.

### 3.3. Photostability Performance of the Liquid-Filled Capsule Formulation

Photostability testing was performed directly on the liquid fill formulation of D-LSD, excluding the capsule shell, to evaluate the intrinsic stabilizing properties of the vehicle. The fill was subjected during 600 s to ICH Q1B-compliant light exposure, and the results were analyzed using LC-UV and LC-IM-MS, focusing on known degradation products previously observed in unformulated solution (Section 3.2.1 and Section 3.2.2).

**Figure 5:**
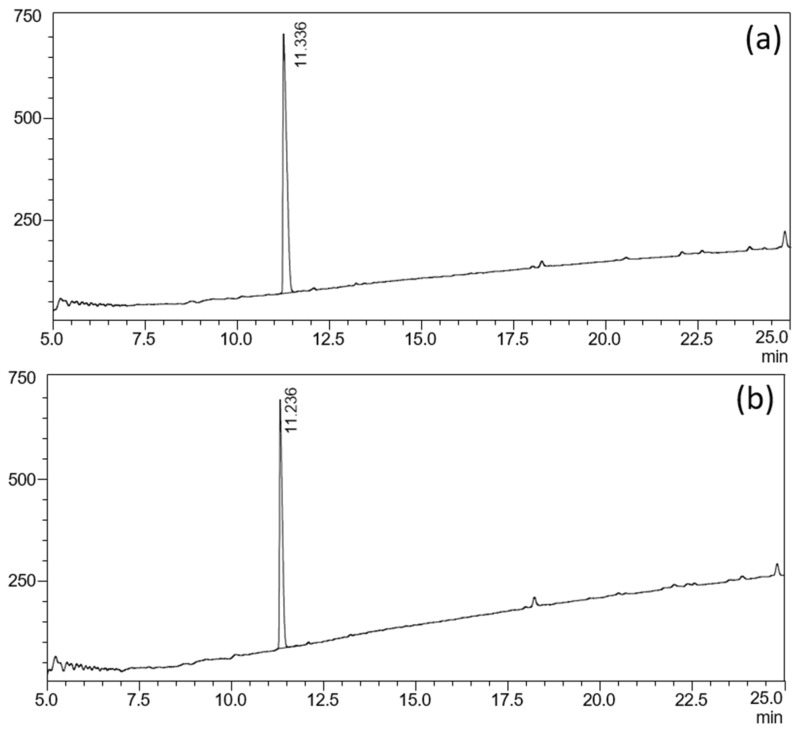
Photostability of the D-LSD liquid fill formulation; (**a**) LC-UV chromatogram of the fill formulation before photostress, showing a single D-LSD peak at 11.2 min; (**b**) LC-UV chromatogram after ICH Q1B light exposure (600 s), with no detectable additional peaks, indicating the absence of degradation. This figure confirms that the liquid vehicle provides effective intrinsic protection against photodegradation.

**Figure 6:**
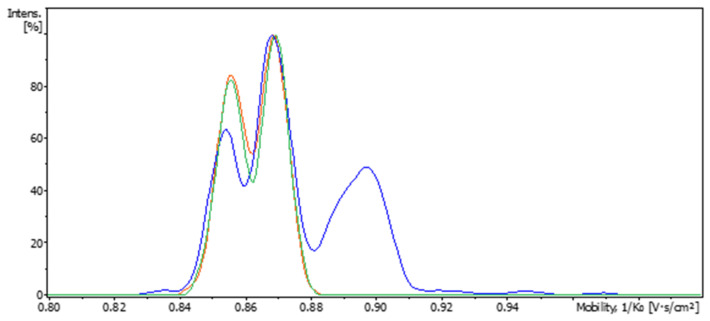
Comparative ion mobility profiles of D-LSD in solution and formulated fill after photostress exposure. Mobilograms recorded at the retention time of the main D-LSD peak (RT 11.2 min) for: a standard solution exposed to simulated light for 120 s (blue trace), the liquid fill formulation exposed for 120 s (green trace), and the same formulation exposed for 600 s (orange trace). No additional drift time features indicative of degradation products (e.g., isomers) were observed in the fill samples, confirming the formulation’s ability to suppress light-induced isomerization under stress conditions.

As shown in Figure 5a,b, the LC-UV chromatograms of the fill formulation before and after light exposure were visually identical. The main peak corresponding to D-LSD remained at 11.2–11.4 min, and no additional peaks appeared at retention times of 8.4 min or 9.5 min, where DP1 and DP2 eluted in the degraded solution. In Figure 6, the overlaid mobilograms recorded at the retention time of the main D-LSD peak (RT 11 min) show the absence of formation of a compound of mobility 0.90 (V.s/cm^2^) in the formulation exposed to light, confirming that the liquid vehicle provides effective intrinsic protection against photodegradation.

These results demonstrate that the vehicle matrix nearly fully suppressed photodegradation under stress conditions that readily triggered transformation in aqueous solution. The stabilizing effect is attributed to the non-aqueous, viscous composition of the fill, which reduces oxygen diffusion and light transmission, while dispersing D-LSD in a low-polarity solvation environment. This minimizes exposure of photo- and redox-sensitive moieties, preventing activation of the pathways that lead to isomerization and oxidation.

Additionally, propylene glycol may exert a secondary protective role as a radical scavenger, limiting the impact of any reactive oxygen species generated upon light exposure. The absence of water also reduces hydrolytic risks, further contributing to chemical stability.

Although the data supports a photoprotection of D-LSD in the fill, the investigational product will consist of opaque liquid filled capsules packed in amber glass containers to provide equal or greater protection with respect to light. The container label will contain the mention “Protect from light”. This multitiered stabilization strategy, combining both physical and chemical barriers, is well-suited for microdosed clinical applications, where compound integrity and dose reproducibility are critical.

The primary degradation products identified under photostress (DP1 and DP2) were characterized using LC-IM-MS and supported by theoretical calculations. While their precise pharmacological or toxicological profiles are not fully established, structurally similar compounds such as iso-LSD and lumi-LSD have been reported to exhibit lower potency and altered receptor activity without clear toxic effects at therapeutic or sub-therapeutic levels. Given the extremely low dose and the absence of novel degradation products in the formulation, the risk of adverse pharmacological impact is considered minimal. Nonetheless, these degradation products have been flagged for inclusion in our broader nonclinical safety program to ensure comprehensive evaluation during clinical development.

To provide a consolidated view of the photostability behavior observed under different experimental conditions, a summary comparison is presented in Table 2. This table contrasts the degradation profile of unformulated D-LSD solution with the results obtained for the liquid fill formulation, highlighting the analytical signatures of each condition and the inferred mechanisms of protection. While the capsule itself was not tested under light exposure in this study, its contribution is discussed based on the expected cumulative protective effect of the multi-tiered formulation design.

Throughout the degradation studies, both in the liquid fill, several degradation products were identified; however, lysergic acid, the expected hydrolysis product of LSD, was never detected based on targeted ion extraction analyses. This targeted approach aligns with the risk-based, phase-appropriate expectations outlined in early-phase pharmaceutical development frameworks, where identifying and controlling the most relevant degradation pathways is prioritized prior to full ICH-compliant stability profiling.

This study specifically addresses the most critical degradation risk for D-LSD: photoinstability. Given its extreme light sensitivity, we prioritized ICH Q1B-compliant testing to evaluate formulation stability during manufacturing and early handling. Broader aspects of robustness, including oxidative degradation and excipient compatibility, are being evaluated as part of ongoing ICH Q1A stability studies at both long-term (2–8 °C) and accelerated (25 °C) conditions. 

## 4. Conclusions

This study addresses the key challenge of D-LSD photoinstability by developing a non-aqueous, liquid-filled capsule formulation.

Using orthogonal analytical and theoretical methods, we identified primary photodegradation products and confirmed that the formulation prevents their formation under ICH Q1B conditions, without introducing new degradation pathways. The full capsule also demonstrated photostability, while long-term and accelerated studies are ongoing under ICH Q1A. The solution-based system ensures dose uniformity, and all excipients are approved for oral use.

These results support the formulation’s inclusion in a multicenter clinical trial and its qualification as an investigational product under the associated IMPD.

## Figures and Tables

**Table 1:** Relative composition of the liquid fill and quantities per a 100 µg capsule.

Ingredients	Function	Relative Composition (% *w*/*w*)	Quantities per Capsule
LSD tartrate	Active pharmaceutical ingredient	0.128%	0.1 mg
Tween 80	Co-solvent and capsule protector	71.144%	55.557 mg
Propylene glycol	Solvent	17.71%	13.89 mg
Purified water	Co-solvent	10.662%	8.333 mg
Sodium acetate trihydrate	Buffering agent	0.356%	0.277 mg

**Table 2:** Summary of D-LSD photostability outcomes in solution vs. formulated fill and capsule.

Test Condition	Main Observations	Detected Degradation Products	Analytical Signatures	Interpretation
Unformulated solution	Rapid degradation under ICH Q1B photostress	DP1 (photoisomer) DP2 (photoxidized) iso-LSD	-D-LSD: RT 11.2 min, drift time 0.85–0.88, *m*/*z* 324.207 -DP1: RT 8.4 min, drift time ~0.885, *m*/*z* 324.207 -DP2: RT 9.5 min, λ shoulder ~275 nm, *m*/*z* 342.202 -iso-LSD: RT 11.2 min, drift time ~0.9, *m*/*z* 324.207	LSD undergoes photoisomerization and oxidation in aqueous solution
Liquid fill formulation (no capsule)	Chemically stable after direct photostress exposure	None detected	No new LC-UV or LC-IM-MS peaks; D-LSD profile retained	Vehicle matrix suppresses photoreactivity via low water activity, viscosity, and solvation effects
Finished capsule (expected)	Not tested directly in this study—included in amber vial	—	—	Expected to provide even greater protection via multi-tier barrier (formulation + capsule + vial)

## Data Availability

Data is contained within the article or Supplementary Materials.

## Supplementary Materials

The following supporting information can be downloaded at: https://www.mdpi.com/article/10.3390/pharmaceutics17060767/s1, Table S1: Detailed parameters of mass spectrometry conditions; Figure S1: LC-MS mass spectra of D-LSD; Figure S2: LC-MS mass spectra of DP2; Figure S3: LC-IM-MS^2^ mass spectra of D-LSD; Figure S4: Fragmentation pattern of D-LSD; Figure S5: LC-IM-MS^2^ mass spectra mass spectra of DP2; Figure S6: Fragmentation pattern of DP2; Figure S7: Mobilogram of DP1.

## References

[1] Burnette E.M., Nieto S.J., Grodin E.N., Meredith L.R., Hurley B., Miotto K., Gillis A.J., Ray L.A. (2022). Novel Agents for the Pharmacological Treatment of Alcohol Use Disorder. Drugs.

[2] Sliedrecht W., De Waart R., Witkiewitz K., Roozen H.G. (2019). Alcohol Use Disorder Relapse Factors: A Systematic Review. Psychiatry Res..

[3] Alper K., Dong B., Shah R., Sershen H., Vinod K.Y. (2018). LSD Administered as a Single Dose Reduces Alcohol Consumption in C57BL/6J Mice. Front. Pharmacol..

[4] Elsilä L.V., Harkki J., Enberg E., Martti A., Linden A.-M., Korpi E.R. (2022). Effects of Acute Lysergic Acid Diethylamide on Intermittent Ethanol and Sucrose Drinking and Intracranial Self-Stimulation in C57BL/6 Mice. J. Psychopharmacol..

[5] Krebs T.S., Johansen P.-Ø. (2012). Lysergic Acid Diethylamide (LSD) for Alcoholism: Meta-Analysis of Randomized Controlled Trials. J. Psychopharmacol..

[6] Maclean J.R., Macdonald D.C., Byrne U.P., Hubbard A.M. (1961). The Use of LSD-25 in the Treatment of Alcoholism and Other Psychiatric Problems. Q. J. Stud. Alcohol..

[7] Niwaguchi T., Inoue T. (1971). Photodecomposition of Lysergic Acid Diethylamide (LSD). Proc. Jpn. Acad..

[8] Walker E.B., McNall S.J., Mansour T.E. (1983). Photoreactivity of Lysergic Acid Diethylamide and Its Possible Utility as a Photoaffinity Labeling Reagent. Biochem. Pharmacol..

[9] Li Z., McNally A.J., Wang H., Salamone S.J. (1998). Stability Study of LSD Under Various Storage Conditions. J. Anal. Toxicol..

[10] Okada Y., Ueno K., Nishiwaki N., Nishimura T., Segawa H., Yamamuro T., Kuwayama K., Tsujikawa K., Kanamori T., Iwata Y.T. (2024). Identification of 1-(Thiophene-2-Carbonyl)-LSD from Blotter Paper Falsely Labeled “1D-LSD”. Forensic Toxicol..

[11] Naomi Oiye É., José Ipólito A., Firmino De Oliveira M. (2017). Quantification of LSD in Seized Samples Using One Chromatographic Methodology for Diode Array Detection and Electrochemical Detection. Forensic Sci. Criminol..

[12] Da Cunha K.F., Kahl J.M.M., Fiorentin T.R., Oliveira K.D., Costa J.L. (2022). High-Sensitivity Method for the Determination of LSD and 2-Oxo-3-Hydroxy-LSD in Oral Fluid by Liquid Chromatography–tandem Mass Spectrometry. Forensic Toxicol..

[13] Grumann C., Henkel K., Stratford A., Hermanns-Clausen M., Passie T., Brandt S.D., Auwärter V. (2019). Validation of an LC-MS/MS Method for the Quantitative Analysis of 1P-LSD and Its Tentative Metabolite LSD in Fortified Urine and Serum Samples Including Stability Tests for 1P-LSD under Different Storage Conditions. J. Pharm. Biomed. Anal..

[14] Žuvela P., Skoczylas M., Jay Liu J., Bączek T., Kaliszan R., Wong M.W., Buszewski B. (2019). Column Characterization and Selection Systems in Reversed-Phase High-Performance Liquid Chromatography. Chem. Rev..

[15] EMA ICH Q1B Photostability Testing of New Active Substances and Medicinal Products—Scientific Guideline. https://www.ema.europa.eu/en/ich-q1b-photostability-testing-new-active-substances-medicinal-products-scientific-guideline.

[16] Wu Q., Wang J.-Y., Han D.-Q., Yao Z.-P. (2020). Recent Advances in Differentiation of Isomers by Ion Mobility Mass Spectrometry. TrAC Trends Anal. Chem..

[17] EMA (2018). EMA/CHMP/190743/2016 Information for the Package Leaflet Regarding Polysorbates Used as Excipients in Medicinal Products for Human Use 2018.

[18] EMA (2017). EMA/CHMP/704195/2013 Propylene Glycol Used as an Excipient 2017.

