# Supplementary material for: Stability-Guided Formulation of a Light-Sensitive D-LSD Capsule for Clinical Investigation
Source: Pharmaceutics. 2025 Jun 11;17(6):767. doi: 10.3390/pharmaceutics17060767 (PMC12196330; doi:10.3390/pharmaceutics17060767)
Supplement: Supplementary file 1 [file pharmaceutics-17-00767-s001.zip › pharmaceutics-3658830-supplementary.pdf]

Supplementary information

Stability-Guided Formulation of a Light-Sensitive D-LSD Capsule for Clinical Investigation

Bernard Do [1,2], Luc Mallet [3,4,5], Maxime Annereau [1,2], Danielle Libong [6,7], Audrey Solgadi [7], Florence Vorspan [8,9], Muriel Paul [10] and Philippe-Henri Secretan [11,*]

1 Clinical Pharmacy Department, Gustave Roussy Cancer Campus, 94805 Villejuif, France
2 Institut des Sciences Moléculaires d'Orsay, CNRS, Université Paris Saclay, 91400 Orsay, France
3 Université Paris-Est Créteil, DMU IMPACT, Département Médical-Universitaire de Psychiatrie et d'Addictologie, Hôpitaux Universitaires Henri Mondor-Albert Chenevier, Assistance Publique-Hôpitaux de Paris, 94000 Créteil, France
4 Sorbonne Université, Institut du Cerveau-Paris Brain Institute–ICM, Inserm, CNRS, 75013 Paris, France
5 Department of Mental Health and Psychiatry, Global Health Institute, University of Geneva, 12011 Geneva, Switzerland
6 Lip(Sys)2—Chimie Analytique Pharmaceutique, Université Paris-Saclay, 91400 Orsay, France
7 UMS-IPSIT SAMM Facility, Université Paris-Saclay, Inserm, CNRS, Ingénierie et Plateformes au Service de l'Innovation Thérapeutique, 91400 Orsay, France
8 Université Paris Cité, INSERM UMRS 1144 Optimisation Thérapeutique en Neuropharmacologie, 75006 Paris, France
9 APHP, GHU NORD, Hôpital Fernand Widal, Département de Psychiatrie et de Médecine Addictologique, 75010 Paris, France
10 Department of Pharmacy, Henri Mondor Hospital, AP-HP, 94000 Créteil, France
11 Matériaux et Santé, Université Paris-Saclay, 91400 Orsay, France
* Correspondence: philippe-henri.secretan@universite-paris-saclay.fr

Table of content

Table S1: Detailed parameters of mass spectrometry conditions 3
Figure S1: LC-MS Mass spectra of D-LSD........ 4
Figure S2: LC-MS Mass spectra of DP2 4
Figure S3: LC-IM-MS² mass spectra of D-LSD 5
Figure S4: Fragmentation pattern of D-LSD 5
Figure S5: LC-IM-MS² mass spectra mass spectra of DP2 6
Figure S6: Fragmentation pattern of DP2 6
Figure S7: Mobilogram of DP1 7

Table S1: Detailed parameters of mass spectrometry conditions

**ESI + - Source**	
Scan range	20-1000 m/z
End Plate Offset	500 V
Capillary	4500 V
Nebulizer	2.2 bar
Dry gas	8 L/min
Dry temp	220°C
**Transfer**	
Deflection 1 delta	60 V
Funnel 1 RF	250 Vpp
Funnel 2 RF	200 Vpp
isCID energy	0 eV
Multipole RF	200 Vpp
**Quadrupole**	
Ion energy	5.0 eV
Low Mass	60 m/z
**TIMS**	
1/k0	0.45 – 1.54 V.s/cm²
Ramp Time	300 ms
ICC target	7.5 Mio
Δt1 deflection transfer - > Cap Exit	-20 V
Δt2 deflection transfer - > deflection discard	-120 V
Δt3 Funnel 1 -> deflection transfer	50 V
Δt4 Accu trap -> Funnel 1 in	50 V
Δt5 Accu exit -> Accu transfer	0 V
Δt6 Ramp start -> Accu exit	20 V
Funnel 1 RF	300 Vpp
Collision cell in	140 V
**TOF**	
Transfer time	65 µs
Pre-pulse storage	3 µs
**MS/MS PASEF**	
Nb of PASEF MS/MS scans	2
Isolation mass range	20-1300 m/z
Isolation width	2-8 m/z
Target intensity	4000
Intensity threshold	100
Collision energy	20 eV

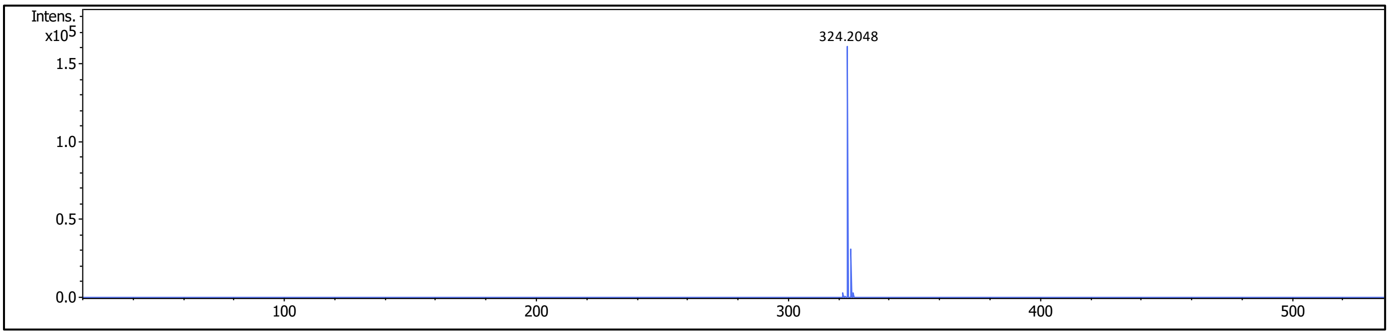

Figure S1: LC-MS Mass spectra of D-LSD

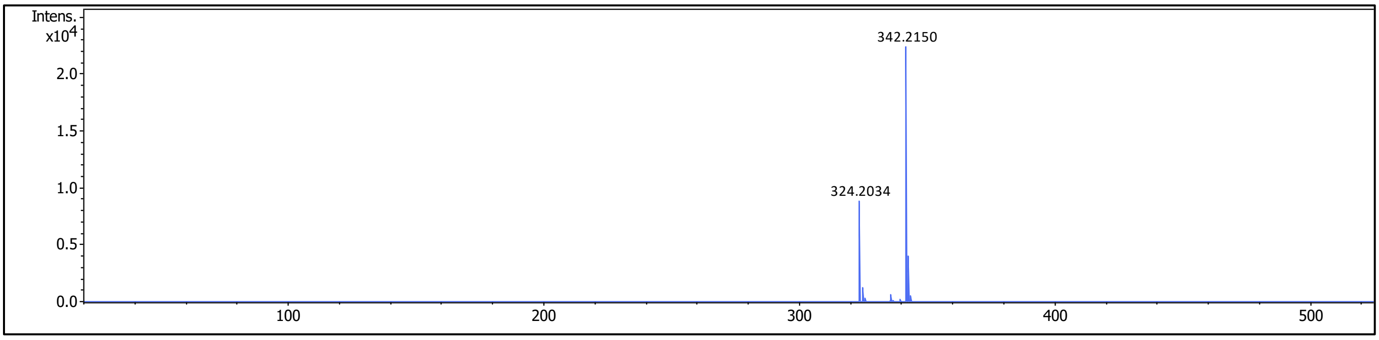

Figure S2: LC-MS Mass spectra of DP2

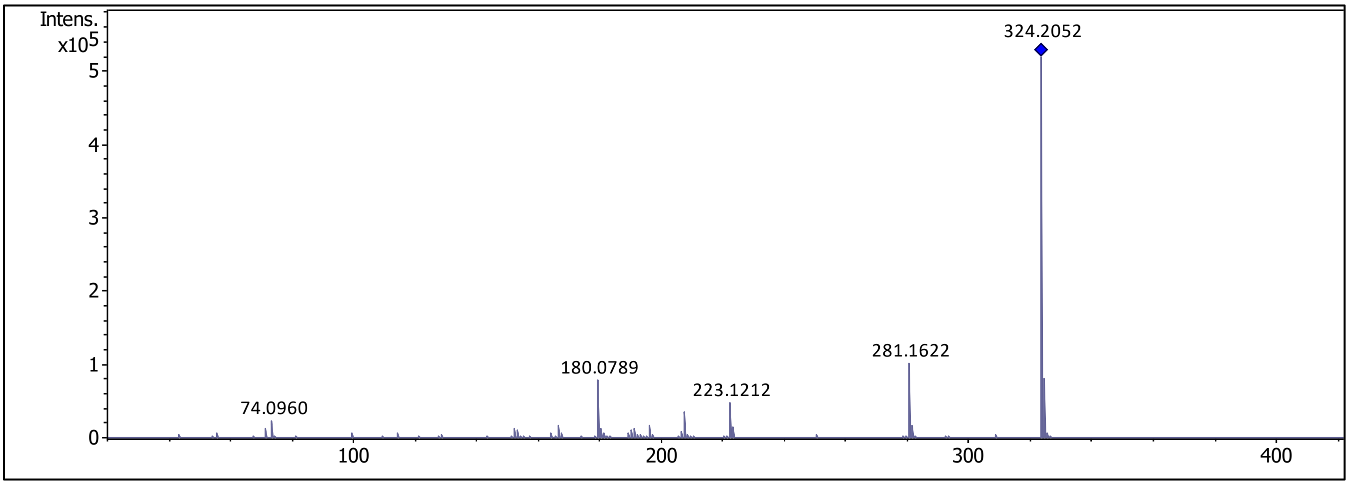

Figure S3: LC-IM-MS² mass spectra of D-LSD

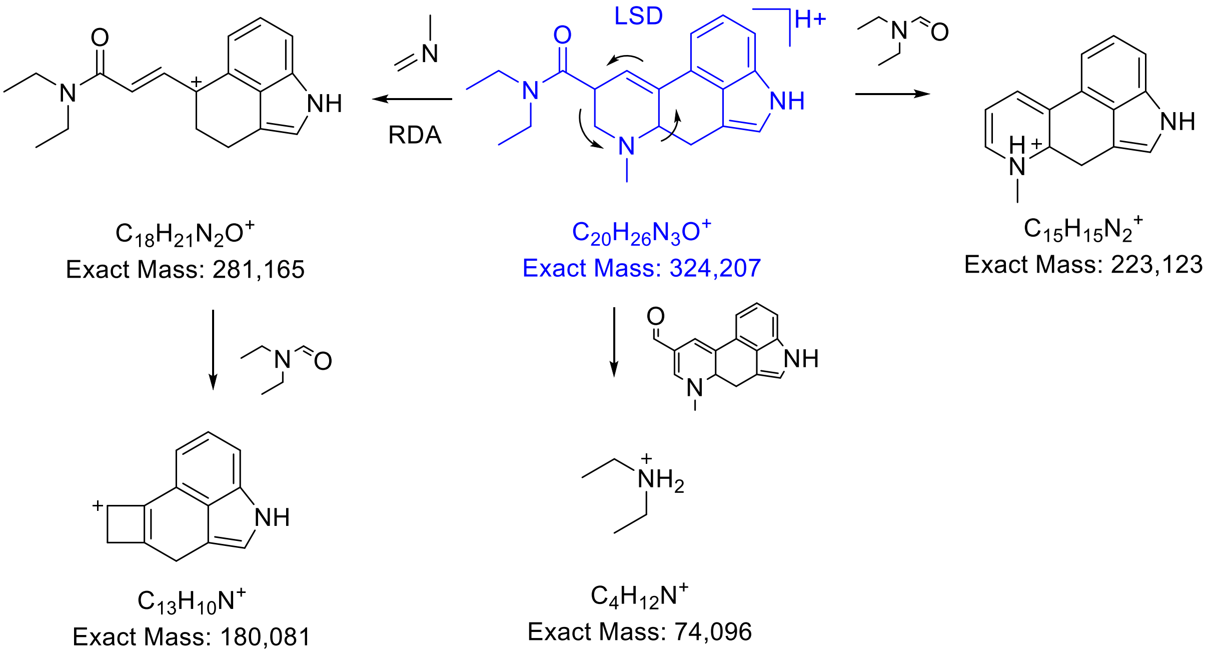

Figure S4: Fragmentation pattern of D-LSD

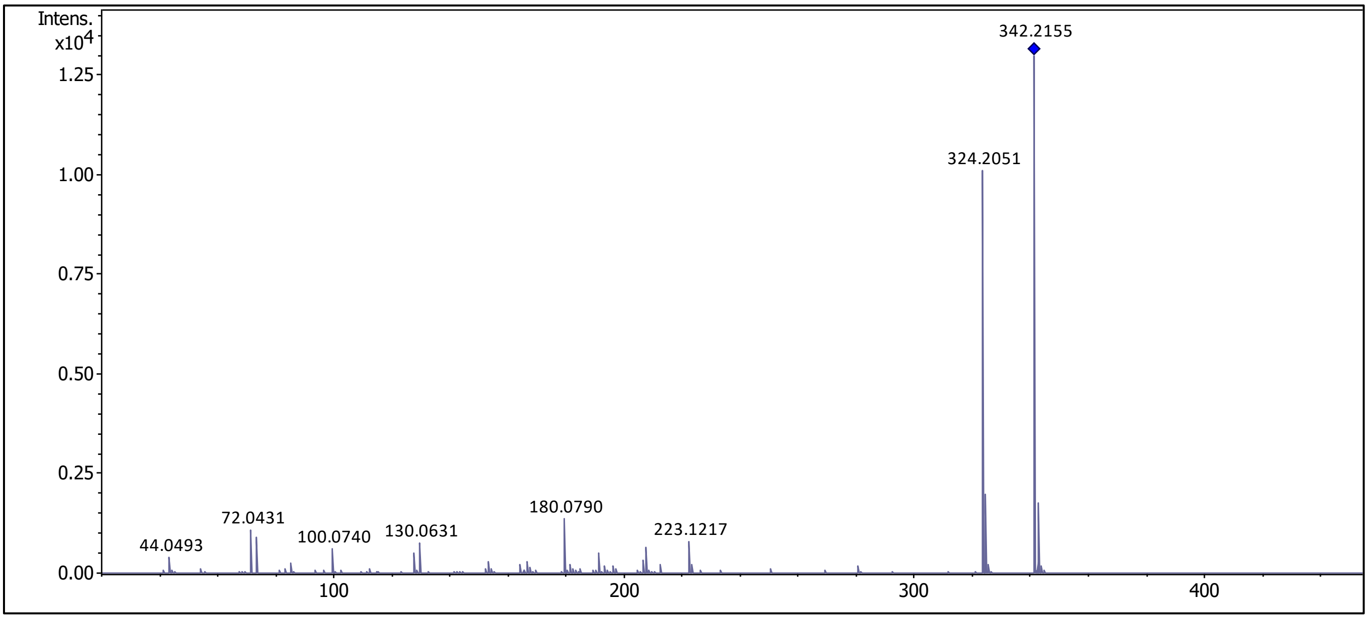

Figure S5: LC-IM-MS² mass spectra mass spectra of DP2

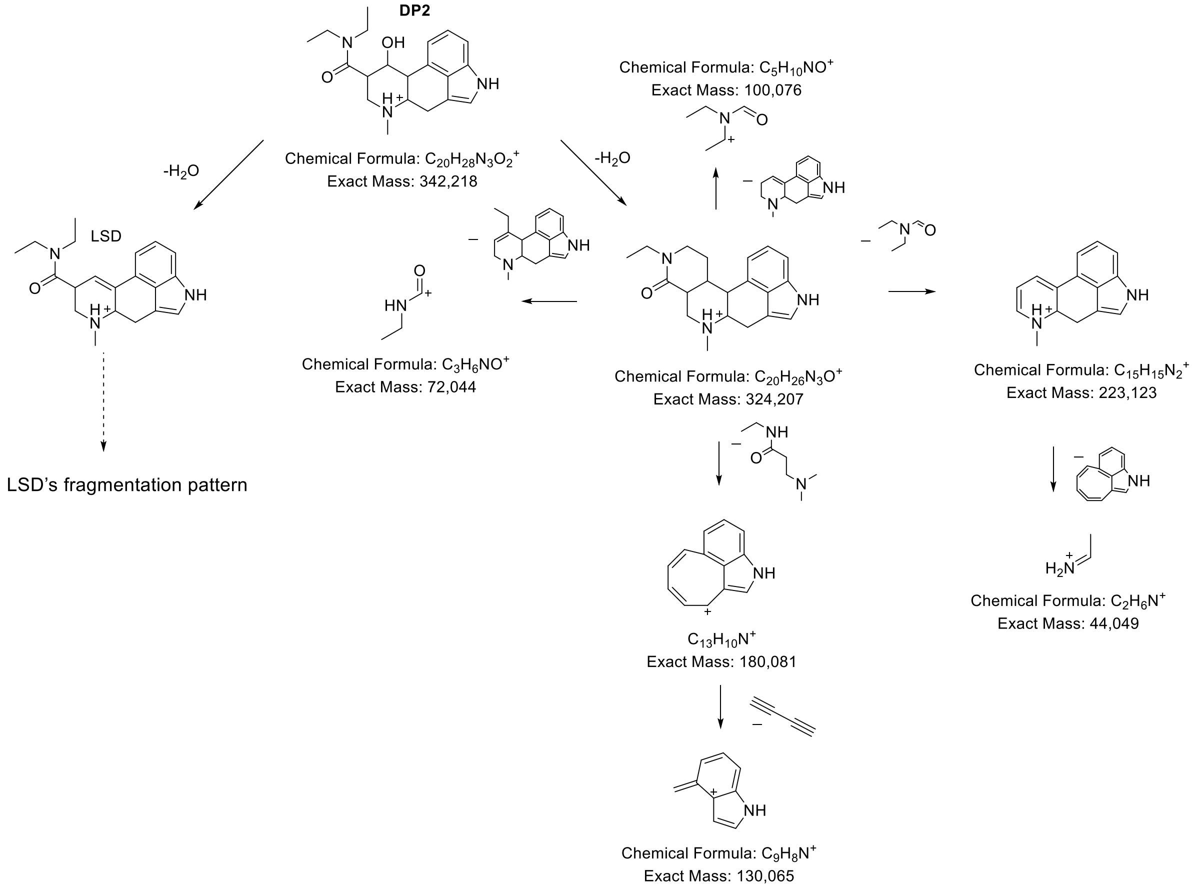

Figure S6: Fragmentation pattern of DP2

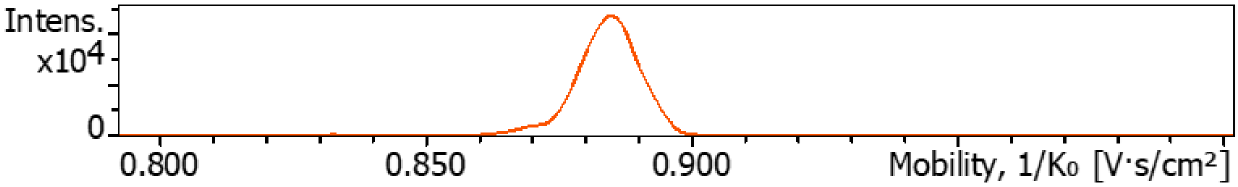

Figure S7: Mobilogram of DP1
